# Outcomes in orthopedics and traumatology: translating research into practice

**DOI:** 10.1590/1413-78522014220601009

**Published:** 2014

**Authors:** Vinícius Ynoe de Moraes, Paula Martins de Oliveira Ferrari, Guilherme Conforto Gracitelli, Flávio Faloppa, João Carlos Belloti

**Affiliations:** 1. Universidade Federal de São Paulo, Department of Orthopedics, São Paulo, Brazil, Department of Orthopedics - Universidade Federal de São Paulo (UNIFESP), São Paulo, Brazil

**Keywords:** Orthopedics, Knee joint, Platelet-rich plasma, Evidence-based medicine, Outcomes research

## Abstract

Clinical research is focused in generating evidence that is feasible to be applicable to practitioners. However, translating research-focused evidence into practice may be challenging and often misleading. This article aims is to pinpoint these challenges and suggest some methodological safeguards, taking platelet-rich plasma therapies and knee osteochondral injuries as examples. Studies and systematic reviews involving the following concepts will be investigated: clinically relevant outcomes, systematic errors on sample calculation, internal and external validity. Relevant studies on platelet-rich plasma for muscle-tendon lesions and updates on osteochondral lesions treatment were included in this analysis. Authors and clinicians should consider these concepts for the implementation and application of dissemination of the best evidence. Research results should be challenged by a weighted analysis of its methodological soundness and applicability. **Level of Evidence V, Therapeutic Studies - Investigating the Results of Treatment.**

## INTRODUCTION

Outcome is defined as the final event of an intervention (of an experimental study) or of an observation (of an observational study).^1 ^Currently, relevant outcome is understood as that effect is important to the patient, such as function and/or quality of life.^2^^,^ 3

It is known that these are the best parameters to translate treatment effectiveness, because they consider the evaluation - subjective and reported by the patient - as the most relevant to health care.^2^ On orthopedics research, it is a surgeon task to decide which outcome is relevant for the studied clinical condition, despite this decision is often a challenge.

The defiance about the choice of the best measure for outcome - that results in an applicable evidence on daily practice (external validity)^4^ is grounding extensive surgical clinical research. Numerous publications devoted to "research on research", example of metalanguage applied to medical science, are dedicated to solving the translational gap between ideal environment research (effective results) and clinical practice (actual results).^5^^-^ 7

However, systematic reviews and evidence-based guidelines are redundant to show that part of the current research does not address the relevant outcomes to clinical practice, resulting in waste of human and economic.^8^^-^ 10 In this context, the ethical implications of conducting research without relevance or optimization of objectives may not be forgotten.^11^^-^ 13

Since the 90's, orthopedic-researchers aim at a paradigm shift from the "surgeon centered" to the "patient-centered" research.^14^^-^ 17 To make it possible, prolific efforts sedimented methodologies (e.g. randomization and blinding methods, intention to treat analysis) and tools (e.g. questionnaires and scales) which are *sine qua non* conditions for enthusiasts of based on evidences orthopedics.^14^^,^ 17 ^-^ 19

In the 2010's, making a research following these above concepts is to ratify the position sedimented by those pioneer researchers. Although the concept is well accepted in the scientific community, there are obstacles and pitfalls regarding methodology and its interpretation.^18^

This review aims to expose concepts, applied to the orthopedic traumatology reality, of clinical research centered in relevant results, with the patient as the main informant of his condition.

### Paradigm shift: prioritizing patient-centered assessment

It is a consensus that clinical outcomes, such as range of motion, strength and bone healing do not reflect entirely the post intervention status and often led the orthopedic community to misguided conclusions.^2^ The more complete concept takes into account the specific functional dimension (clinical condition or region) and / or overall quality of life (physical, mental and social aspects).^5^^,^ 20

Subjectively, let the patient judge his own condition leads us to avoid two major pitfalls: the interventor bias, evaluating their own interventions and their subsequent judgment of success or failure.

As additional condition for the choice of a good outcome, attention should be given to the establishment of a robust method (internal validity) of hypothesis verification, what could increase the results applicability (external validity). One should opt for conducting the study so that it can be the most reliable conditions of daily life, so that its effectiveness is proven. Some randomized clinical trials are often targets of criticism when conducted "very controlled" because their results can translate more efficiency than effectiveness. (Table 1)

**Table 1:** Concepts for methodological assessment.

Concept^3,5^	Definition
Internal validity	Ability of a study to reduce the chance of systematic errors (bias) through the optimization of the research methods
External validity	It is the ability of the result to generate reliable generalizations to its target population (applicability)
Effectiveness	Ability of an specific intervention to produce the expected results - used under normal circumstances (medical practice, for example)
Efficiency	Ability of an specific intervention to produce the expected results - when used under ideal circumstances (laboratory studies, clinical studies with very restricted methodology)

### How to measure these results? Sample calculation and clinical tools

For the evaluation of patient focused outcomes, a large number of general and specific clinical tools are available and validated for our population. (Table 2)

**Table 2:** Necessary characteristics to a questionnaire as a research tool.

Characteristics^2,5^	Description
Reproducibility (test-retest reliability)	Ability of different measurements to generate the same result under steady and constant conditions
Validity	Ability of a test to measure what it proposes. It is subdivided into validity of: 1. Contents: Subjective evaluation of whether the components of the test include content / size to be measured; 2. Criterion: comparative evaluation against established "gold" standard; 3. Constructive: Assessment if the components of the test are comparable to clinical parameters and / or laboratory parameters relevant to clinical condition, in the absence of a gold standard.
Responsiveness	Ability of a test to demonstrate relevant differences to researchers, surgeons and patients
Specificity	Ability to demonstrate plausible differentials to the studied condition

The clinical tools allow considering the patient in the trial of clinical interventions and strengthen the actions of the researcher in evaluating what treatment method is the best. As a principle, it is recommended the use of specific tools (organ/member/specific disease) instead of the general ones.^5^

As a complement, the practical use of these concepts, arises the concept of minimally significant clinical difference (MSCD), defined in 1989.^21^ The initial argument of its practical plausibility is the finding that frequently identified statistic differences do not reflect relevant clinical differences.^2^^,^ 21 The definition of MSCD is set up as:^22^ "the smallest difference in a punctuation / score in the domain of interest in which patients perceive the presence of benefit and would require a shift in treatment paradigm, in the absence of adverse side effects and excessive costs."

### Translation of the outcomes: clinically relevant differences

Clinical trials may include one single group or more than one group. When there is a comparison, there is a possibility of generating assertions regarding the effectiveness of an A intervention versus a B intervention. To allow this it is necessary to be sure that both groups are comparable, often randomly allocated.^23^

Randomization, in itself does not guarantee that the comparison between the groups is valid.^24^ Statistically, it is necessary that the groups present sufficient sample number, ideally calculated a priori. The challenges of this step are described in Table 3.

**Table 3:** Sample number determination - difficulties and challenges.

Research scenario	Characters
Insufficient sample number, difference detected between groups	The difference between the groups may have occurred at random, unreliable results. Frequent in unplanned subgroup analyzes *. TYPE I ERROR.
Insufficient sample size, no difference detected between the groups	The difference between groups was not detected because there are few individuals in the comparison groups. Increasing the sample size will detect these differences. Frequent scenario in surgery studies. TYPE II ERROR.
Sufficient sample size, calculation performed previously	Results are possibly extrapolated to clinical practice. Steps of evidence-based medicine should be applied to ensure the reliability of the final product. (1. Clinical question; 2. Searching the best evidence; 3. Critical analysis; 4. Application) There is the possibility to detect statistical differences without clinical relevance (see next topic).

Researchers often opt for subgroups analysis. The data obtained should be considered with caution.

### Applied clinical research: platelet-rich plasma

Until present times, studies involving platelet rich plasma failed to demonstrate the effectiveness of this treatment modality for orthopedic conditions such as rotator cuff tears, lateral epicondylitis and knee ligament injuries.^25^ These studies - due to their recentness - are already focused on self-reported functional outcomes, under the presented research protocol. Many of these, despite being presented with sophisticated research designs are research products with low sample number, which may lead the reader to reach erroneous conclusions. Furthermore, the concept of minimally significant clinical difference must be taken into account.

Meta-analysis involving patients who underwent application of platelet rich plasma demonstrated a statistical difference for the pain outcome (measured by analogue scale) for arthroscopic rotator cuff repair, lateral epicondylitis, tendinopathy and rupture of the Achilles tendon. In this result - evaluated for up to three months, it was identified that the difference between the averages for allocation groups was - 0.95 with a confidence interval (95% CI) going from the minimum to maximum of -1.41 -0.48.

Surgeons and researchers should reflect if a maximum difference, in the most favorable scenario to Platelet-Rich Plasma of 1.41 brings benefit to justify the application of Platelet-Rich Plasma. In this meta-analysis, the authors indicate that this difference is marginal and that possibly does not translate into relevant clinical benefit.^25 ^In this research spectrum, studies show that differences above 3 points are those that result in relevant clinical differences.^2^^,^ 26

### Applied clinical research: osteochondral knee lesions

The incidence of osteochondral lesions (OCL) of the knee are estimated at 900,000 per year in the United States of America^27^ and affect the population in different age groups with 36% greater prevalence in the population of athlets.^28^ The low capacity for regeneration of cartilage tissue, associated with frequent progression to osteoarthritis make these lesions widely studied and of great clinical interest issue currently.

When studying OCL in clinical trials, we must have in mind some confounding variables included in several clinical trials that are usually associated to patients with OCL, but should not be considered all together. An example that is frequently reported is the analysis on the anterior cruciate ligament (ACL) intervention associated with OCL,^29^^,^ 30 in which the treatment with ACL reconstruction can directly influence the results of cartilage repair and vice versa.

Osteochondritis dissecans (OCD) is another greatly mentioned theme in clinical trials for treatment of cartilage injuries.^31^ Given the different behavior of this disease compared to isolated cartilage lesion, this patient population should be assessed separately in the studies.

What if we do not get a significant sample to study the isolated desired population? In these cases the ideal is to create subgroups in the same analysis in order to isolate the effect of each population. An example of a clinical trial is: Micro-fracture versus mosaicoplasty in isolated chondral injury and OCD. When assaying these patients, analysis of groups for isolated chondral injury and OCD should be separated.

The main or primary outcomes in LOC clinical studies should be the patient's function, quality of life and treatment complications. Functional outcomes are assessed by functional scores - among which the most used are the Western Ontario and McMaster Universities questinnaire-WOMAC,^32^ the International cartilage repair score-ICRS SCORE, the Knee injury and osteoarthritis outcomes score-KOOS,^33^ the International knee documentation Committee subjective knee evaluation form - IKDC^34^ and the Lysholm knee scoring scale-LYSHOLM^35 ^- some presented on Table 4.^36^^-^ 41 The quality of life can be assessed by the SF-36^42 ^or World Health Organization (WHOQoL) questionnaires. For treatment complications such as infection and stiffness, revision surgery should also be considered among the most important outcomes.

**Table 4:** Instruments for functional scores assessment.

Instrument	Description
DASH (Disability of arm, shoulder and hand)	Region-specific questionnaire, self-applied. Translated and validated into Brazilian Portuguese, in a population with rheumatoid arthritis. Measures dysfunction of the arm, shoulder and hand. Its evaluation considers activity of both upper limbs, globally. Has additional (optional) modules addressing sports, music and work performance. There is good correlation between full version and summarized version (Quick DASH).
PRWE (Patient-rated wrist evaluation score)	Region-specific questionnaire, self-applied. Translated into Brazilian Portuguese. It still has to be validated. Initially idealized for distal radius fractures, measures dysfunctions of the affected wrist. Approaches pain and function. There are studies demonstrating good psychometric qualities. Adequate correlation with SF-36 and DASH.
CONSTANT – MURLEY (Constant-Murley questionnaire)	Region-specific questionnaire, applied by the interviewer. Initially indicated for all shoulder conditions; however, there was the development of disease-specific scores, such as WORC (for the rotator cuff) and ROWE (for instability). It assesses pain, everyday life activities, strength and range of motion. Studies show good reproducibility, despite it lacks specificity for shoulder instability.
MHQ (Michigan hand questionnaire)	Region-specific questionnaire, self-applied. Indicated for general assessment of all conditions of the hand. Evaluates pain, function, esthetic and satisfaction. Unlike the DASH questionnaire, it rates separately left and right hands.
BHQ (Boston Carpal Tunnel Questionnaire, Levine-Katz Questionnaire)	Disease-specific questionnaire self-applied or applied by the interviewer. Evaluates function and symptoms. There is extensive literature validating this tool, with good correlation with the SF-36 and DASH. Indicated for evaluation of patients with carpal tunnel syndrome..
WORC (Western Ontario Rotator Cuff Index)	Disease-specific questionnaire, for rotator cuff evaluation. It is the most used of Western Ontario Shoulder Indexes, which also includes tools for instability (WOSI) and osteoarthritis (WOOS) of the shoulder.
UCLA (University of California at Los Angeles Shoulder Rating Scale )	Region-specific questionnaire, self-reported. Used to assess shoulder function. Evaluates pain, function, range of motion/active flexion, strength/active flexion and satisfaction. The instrument is criticized due to the empirical generation of the questionnaire items, different weighing between the evaluated criteria without a supporting methodological background.
SST (Simple Shoulder Test)	Region-specific questionnaire, self-reported. Used for the evaluation of every shoulder condition of the shoulder. Consists of 12 “yes or no” questions.
WOMAC	Region-specific questionnaire, self-applied. Validated for personal, phone, or electronic interview, through computer or cell phone. Translated and validated into Brazilian Portuguese.^32,36^ Originally developed in 1982 to detect treatment response for osteoarthritis of hip and knee. Currently, it has been used for chondral lesions of the knee and injury of the anterior cruciate ligament (ACL). It is based in three parameters: pain during various movements and positions, severity of joint stiffness and difficulty in performing activities of daily living. The abridged version has been used but is not recommended by the WOMAC web site. The questionnaire is available on the website after request approval (http://www.womac.org).
IKDC (Subjective Knee Evaluation Form)	Region-specific questionnaire, self-applied and not validated for interviews. Translated and validated into Portuguese.^34,37^ Developed for various knee injuries. The IKDC addresses symptoms (pain, stiffness, edema, joint locking and instability) and daily and sports activities, current functions and functions prior to injury (the latter topic is not accounted for the score). Indicated for knee injuries (ACL, anterior cruciate ligament; PCL, posterior cruciate ligament, collateral ligaments, osteochondritis dissecans, knee sprain and meniscal lesion) and corrective interventions (recosntructions of ACL, PCL, and collateral ligaments, meniscal repair, meniscectomy, chondral injury repair, platelet rich plasma infusion, tibial osteotomy and lateral release. Questionnaire available at http://www.sportsmed.org/tabs/research/ikdc.aspx
Tegner	Questionnaire created for interviews, but currently self-applied. Developed in 1985 to assess the level of physical and sports activity of the patients. Originally suggested as a complement to the LYSHOLM score in patients with ACL injury. Based on a range of daily living, recreation and competitive sports activities that are identified to the patient habits. Available on the original publication.^38^
AOFAS	Questionnaire created in 1994 by a committee of the American Orthopedic Foot and Ankle Society (AOFAS). Divides the foot and ankle evaluation based on anatomical scales: hind foot and ankle, mid foot, metatarsal phalangeal (MF) and inter phalangeal (IF) of the hallux, MF and IF the smaller toes, which allows its use in several diseases and interventions of the foot and ankle. Translated in 2008, full text available in the original publication in Portuguese.^39^
Kujala	The Kujala score or scale of the anterior pain of the knee, developed in 1993 is a self-applied questionnaire. It features 13 items evaluated at rest and after specific activities such as walking, running, jumping, squatting, sitting for long periods and climbing stairs. Currently, it is widely used for clinical studies and for monitoring patients with patellofemoral or anterior knee pain. Translated and adapted into Portuguese in 2011.^40^ It is sensitive for anterior knee pain detection, but poor for differentiate recurrent patellar dislocation and single patellar dislocation.
Lysholm	Region-specific questionnaire, self-applied. Validated for personal interview, but frequently used as self-applied. It assesses joint stability after ligament reconstructions. The revised scale has 8 categories: gait, support, joint locking, instability, pain, edema, climbing stairs and squatting. Currently used to assess ligament injuries (ACL, PCL and collateral), meniscal,chondral and knee dislocation. Used to evaluate interventions such as arthroscopy, ligament reconstruction, cartilage repair, tibial osteotomy, infusion of hyaluronic acid and therapeutic exercises. Translated and adapted into Portuguese. Full text publication available.^35,41^

Many clinical trials on cartilage focus on less important end points for clinical studies, such as imaging studies and postoperative biopsies. There is no question on the validity of these analysis for understanding the disease, but the clinical and radiological or clinical histological correlation are often very weak and do not express the functional outcome of these patients.^43^

## FINAL CONSIDERATIONS

The incorporation of the above outlined concepts are important for a good practice of MBE, both from the perspective of the researcher-orthopedist, as of the consumer of health information. It is an essential condition to conduct research in orthopedics and traumatology surgery.
